# Exploratory risk prediction of type II diabetes with isolation forests and novel biomarkers

**DOI:** 10.1038/s41598-024-65044-x

**Published:** 2024-06-22

**Authors:** Hibba Yousef, Samuel F. Feng, Herbert F. Jelinek

**Affiliations:** 1 Biotechnology Research Center, Technology Innovation Institute, Masdar City, P. O. Box 9639, Abu Dhabi, United Arab Emirates; 2 Department of Science and Engineering, Sorbonne University Abu Dhabi, Abu Dhabi, United Arab Emirates; 3 SUAD Research Institute, Sorbonne University Abu Dhabi, Abu Dhabi, United Arab Emirates; 4 Department of Medical Sciences, Khalifa University, 127788 Abu Dhabi, United Arab Emirates; 5 Biotechnology Center, Khalifa University, 127788 Abu Dhabi, United Arab Emirates

**Keywords:** Diabetes, Inflammation, Oxidative stress, Mitochondrial dysfunction, Isolation forest, Predictive modelling, Type 2 diabetes, Machine learning

## Abstract

Type II diabetes mellitus (T2DM) is a rising global health burden due to its rapidly increasing prevalence worldwide, and can result in serious complications. Therefore, it is of utmost importance to identify individuals at risk as early as possible to avoid long-term T2DM complications. In this study, we developed an interpretable machine learning model leveraging baseline levels of biomarkers of oxidative stress (OS), inflammation, and mitochondrial dysfunction (MD) for identifying individuals at risk of developing T2DM. In particular, Isolation Forest (iForest) was applied as an anomaly detection algorithm to address class imbalance. iForest was trained on the control group data to detect cases of high risk for T2DM development as outliers. Two iForest models were trained and evaluated through ten-fold cross-validation, the first on traditional biomarkers (BMI, blood glucose levels (BGL) and triglycerides) alone and the second including the additional aforementioned biomarkers. The second model outperformed the first across all evaluation metrics, particularly for F1 score and recall, which were increased from 0.61 ± 0.05 to 0.81 ± 0.05 and 0.57 ± 0.06 to 0.81 ± 0.08, respectively. The feature importance scores identified a novel combination of biomarkers, including interleukin-10 (IL-10), 8-isoprostane, humanin (HN), and oxidized glutathione (GSSG), which were revealed to be more influential than the traditional biomarkers in the outcome prediction. These results reveal a promising method for simultaneously predicting and understanding the risk of T2DM development and suggest possible pharmacological intervention to address inflammation and OS early in disease progression.

## Introduction

Type II diabetes mellitus (T2DM) is a metabolic disorder characterized by heterogeneous pathophysiology, clinical presentation, and disease progression^1^. With an estimated 463 million people above the age of 20 currently suffering from the disease and a projected increase to 700.2 million by 2045^2^, the rising burden of T2DM and associated comorbidities, including cardiovascular and renal disease, is a serious cause for concern worldwide. Thus, it is of increasing interest to identify methods of early prediction of disease and its progression, as current methods of monitoring HbA1c and blood glucose levels (BGL) have inherent limitations^1^ and result in a proportion of undiagnosed cases in the population as well as lacking specificity for comorbidities^3^. Hence, there is a need to find alternative biomarkers to offer a more comprehensive understanding of pathological processes contributing to disease progression to T2DM.

Early identification of individuals at risk of developing T2DM is a priority for the prevention of long-term disease complications. Lifestyle interventions including dietary changes and exercise for the purpose of weight loss have achieved a 28–58% reduction in diabetes incidence in a safe and cost-effective manner, with pharmacological intervention only required in patients not responding to these interventions^4^. Significant in the development and progression of diabetes are oxidative stress (OS), inflammation, and mitochondrial dysfunction (MD), all of which contribute to insulin resistance and shortage. Furthermore, these three processes induce and exacerbate one another as a result of the interdependence between inflammatory mediators, free radical production, and the mitochondrial electron transport chain, creating a loop that sustains the conditions necessary for T2DM development and progression^5–7^. Given their significance, markers for OS, inflammation and MD have been investigated, mostly individually, as potential biomarkers for early disease detection and prevention^8–10^.

There is a vast amount of research addressing the challenge of early T2DM prediction utilizing machine learning. The dataset most commonly deployed for this purpose is the Pima Indians Diabetes Dataset (PIDD), which contains a total of 8 T2DM predictor variables including age, BMI, BGL, and insulin levels, among others^11^. Another dataset is the Early Classification of Diabetes (ECD)^12^, containing a variety of signs and symptoms associated with the risk of T2DM development. Various other studies have also investigated the use of novel biomarkers such as exhaled breath profile^13^, as well as metabolic profile^14^ and time-series data^15^. However, extensive datasets that include inflammation, OS, and MD biomarkers with respect to diabetes progression are not currently available.

Various challenges exist in the task of T2DM risk prediction^16–19^. Datasets for T2DM prediction, as is common in medical datasets, suffer from class imbalance, given that the global prevalence of T2DM is approximately 6.28%^20^. Hence clinical studies contain more control data with or without complications compared to diabetes data. Most classification algorithms perform poorly on imbalanced datasets. These classification algorithms are often biased towards the majority class, which poses a problem in health-related tasks, given that the cost of missing disease occurrences is often higher than the misclassification of healthy individuals^21^. Popular approaches for mitigating class imbalance include data-level and algorithmic-level methods. Oversampling and under-sampling are commonly employed solutions on the data level; however, they are complicated by overfitting and information loss, respectively^22,23^. On the algorithmic level, misclassification of the minority class can be penalized more heavily through cost-sensitive learning and boosting techniques such as AdaBoost^24^. In Barmparis et al.^25^, the authors utilised the ECD dataset to compare the performance of various machine learning models, including RF, K-nearest neighbors, SVM, and others to predict the risk of T2DM. Synthetic minority oversampling technique (SMOTE) was used to balance the data classes, and the best-performing models were RF and KNN at an accuracy of 0.992. Moreover, SMOTE was also employed by Azad et al.^26^ and ElSeddawy et al.^23^ for class balancing, constructing risk classifiers on PIDD with a decision tree and ANN, respectively, as the models for classification. Another version of SMOTE, namely SMOTETomek, was used by Roy et al.^27^ to address class imbalance in another T2DM risk classifier constructed using an ANN. An additional class balancing method, SMOTENN, was utilised by Feng et al.^28^, combining SMOTE and edited nearest neighbor for minority oversampling. Finally, adaptive synthetic sampling (ADASYN) was employed by Tasin et al.^29^ along with extreme gradient boosting (XGBoost) for early diabetes prediction in Bangladeshi patients.

In cases of extreme class imbalance associated with obtaining and labelling the positive class, one class classification (OCC), also known as anomaly detection, is a useful alternative as it only requires the presence of negative class examples for training the classifier. Essentially, this unsupervised method learns a decision boundary around the target class (inliers) and identifies instances outside of it as anomalies or outliers^30–33^. Anomaly detection has been applied to Alzheimer’s disease diagnosis^34^, identifying acute myeloid leukemia associated genes^35^, and abnormal skin tissue detection^36^. In the realm of T2DM, anomaly detection has been utilised for identifying heterogeneities in diabetes populations for targeted intervention^37,38^, where Fang et al. employed hierarchical clustering and Argaw et al. proposed K-Nearest Neighbors, Isolation Forest (iForest), and One-class SVM for this purpose. For early T2DM risk estimation, iForest was utilised by Fitriyani et al.^39^ for data preprocessing through outlier detection and removal. However, conventional binary algorithms were used to perform the classification task.

Another significant challenge in T2DM risk prediction is the black box nature of many of the proposed models. Black-box models including Random Forest (RF), artificial neural networks (ANN), and support vector machine (SVM) are the most frequently used models for T2DM risk prediction^16–18^. However, they inherently lack interpretability and are rarely explained adequately to assist medical professionals in decision-making^19^. Various studies investigating the prediction of diabetes have incorporated explainability modules, including Shapley additive explanations (SHAP)^29,40,41^ and local interpretable model-agnostic explanations (LIME)^29,41,42^. These studies only incorporated basic clinical and demographic variables such as age, BMI and glucose levels, which are useful for early prediction but do not provide insights for potential targets for the prevention of T2DM. Limited clinical and demographic variables can identify individuals at high risk of developing T2DM but fail to reveal the underlying mechanisms or causal factors contributing to disease onset. Consequently, there is a need to integrate a wider range of biomarkers which could offer deeper insights into the etiology of T2DM and support the formulation of effective prevention strategies.

The main aim of this study was to perform exploratory risk prediction of T2DM with biomarkers of inflammation, OS and MD using OCC in the presence of scarce data. Given that these biomarkers are not routinely assessed as part of standard clinical practice, data availability is a major challenge, particularly the presence of positive samples (patients developing T2DM in this case). Hence, the use of OCC is particularly valuable in this context as it allows for effective modelling and prediction even when positive instances are rare, thereby providing a robust framework for early identification of at-risk individuals despite limited data. Thus, the present work provides a two-fold novelty. First, to our knowledge, the aforementioned biomarkers have not been incorporated in the context of ML, and second, OCC, including iForest, has not been utilised for the task of early T2DM risk estimation.

## Data and methods

### Dataset, participants, and sample collection

**Figure 1:**
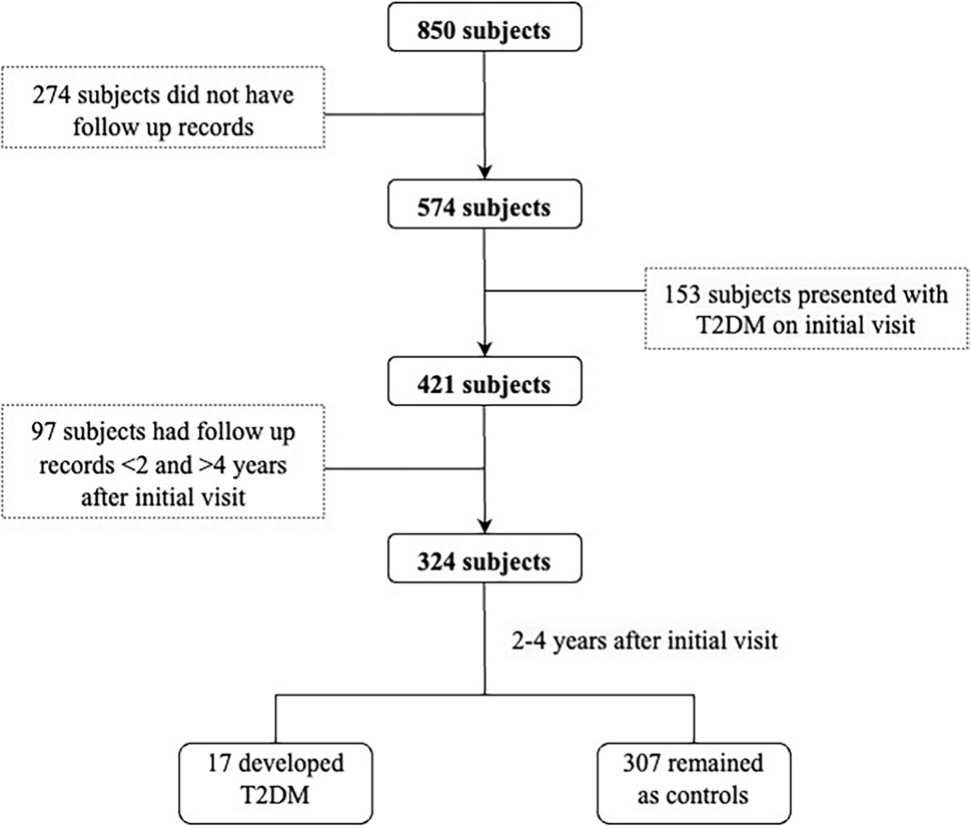
Flowchart for inclusion and exclusion of subjects.

The subjects in this study were attendees of a rural diabetes screening clinic at Charles Sturt University (DiabHealth), Albury, Australia between the years 2002 to 2015. A total of 2716 entries were obtained from 850 patients, with information on more than 180 attributes. Subjects were included if they initially presented without T2DM, and data of a subsequent visit 2–4 years later was available for longitudinal analysis that identified progression to T2DM in a subsection of the cohort. Participants were classified as having developed T2DM if they reported a diagnosis of T2DM, were on glucose-lowering medication or had a fasting BGL ≥ 7 mmol/L following the initial screening 2–4 years prior. Inclusion and exclusion of participants is clarified in Fig. 1.

HbA1c was not considered a diagnostic criterion in our study due to missing values and differing methods used to obtain HbA1c values. Some of the data were obtained as a point of care testing (POCT), and different laboratories were also used for some of the entries, which may pose a problem due to a lack of HbA1c standardization^43,44^. Additionally, HbA1c is sensitive to changes in red blood cell cycles, including vitamin B-12 deficiency, which can produce falsely elevated levels^45^.

Based on these criteria, a total of 324 participants were included, with 17 developing T2DM, and 307 remaining as controls. The incidence is slightly higher than the Australian data in the current cohort, being approximately 5% versus the Australian 0.3%^46^. Body mass index (BMI), blood pressure measurements, and lipid profiles including high-density lipoprotein (HDL), low-density lipoprotein (LDL), triglycerides, and total cholesterol (TC) were measured as detailed in^47^. Blood and urine samples were collected and prepared for measurement of biomarkers of OS, inflammation, MD, and hemostasis according to the methodology detailed in^8^ and^10^. BGL was determined from finger prick POCT. The study was approved by the Charles Sturt University Human Research Ethics Committee. Written informed consent was obtained from all participants prior to sample collection. This research was performed in accordance with the Declaration of Helsinki.

### Data preparation and statistical testing

The dataset presented missing value rates of 5–10%. Missing values were imputed using the mean of data subsets, which were extracted based on selector variables that had the highest Information Gain criterion as detailed in Venkatraman et al.^48^, which provided a complete dataset for this study. Shapiro–Wilk tests confirmed the non-normal distribution of continuous variables and were thus expressed as median (25th percentile, 75th percentile). Spearman correlations were computed between all variables of interest, confirming only weak correlations (|ρ|< 0.6) (see Supplementary Fig. S1 online). Wilcoxon rank-sum and χ^2^ tests were utilised to analyze continuous and categorical variables, respectively, with p-values < 0.05 considered significant. All statistical tests were performed using R-Studio (1.4.1717) and R (4.1.0).

### Predictor variables

A total of 17 features were considered for the predictive modelling, where baseline values were considered 2–4 years prior to glycemic outcome as follows:

- *Inflammatory biomarkers* C-reactive protein (CRP), monocyte chemoattractant protein-1 (MCP), interleukin-6 (IL-6), interleukin-1β (IL-1β), interleukin 10 (IL-10) and insulin-like growth factor (IGF-1).
- *OS biomarkers* 8-isoprostane, 8-hydroxydeoxyguanosine (8-OHdG), and oxidized glutathione (GSSG). GSSG was selected over its reduced form (GSH), as the production of GSH may be ramped up in response to chronic OS^49^ and would therefore not reflect this state accurately.
- *MD biomarkers* humanin (HN) and mitochondrial open-reading-frame of the twelve S rRNA-c (MOTS-c), which are mitochondrial-derived peptides (MDPs), in addition to P66Shc.
- *Hemostasis biomarkers* complement component 5a (C5a) and D-dimer.
- *Traditional features* fasting BGL, BMI, and triglycerides. The value of these three predictors has been shown previously^50–52^, and were therefore considered for this study.

### Isolation Forest (iForest) algorithm

**Figure 2:**
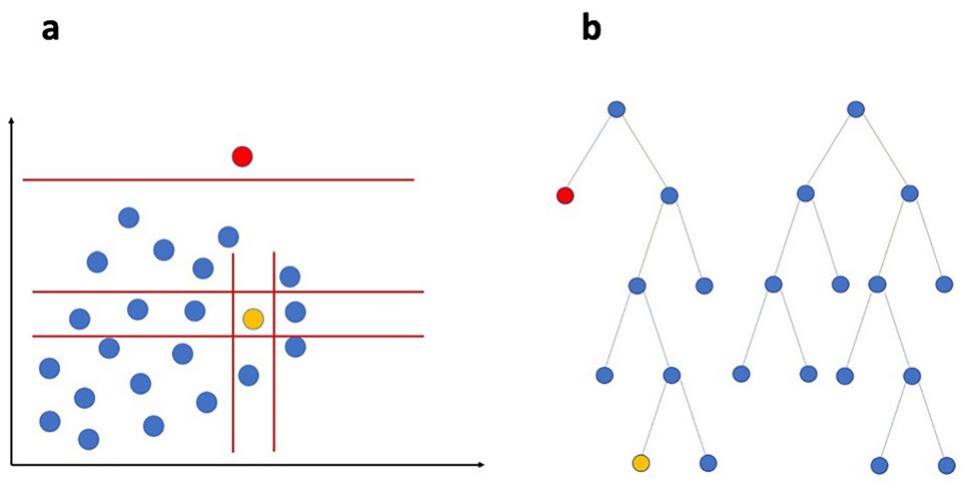
Figures *a* and *b* depict the classification of datapoints using iForest. The yellow point is an inlier, while the red point is an outlier, or an anomaly. (**a**) iForest uses random splits across dimensions in the data, and as depicted, fewer partitions are required to isolate the outlier (red) when compared with the inlier (yellow). In (**b**), the outlier is isolated closer to the root node, requiring a shorter depth or path length.

iForest is an unsupervised, binary tree anomaly detection algorithm developed by Liu et al.^53^. Conceptually, anomalies are those data points that require shorter depths, or path lengths, to be isolated from the majority of other points during successive splitting of the dataset using an ensemble of isolation trees, as can be seen in Fig. 2.

Given n points, the anomaly score s for point x can be calculated using the following equation:

1 $$s\left( {x,n} \right) \, = 2^{{ - \frac{{E\left( {h\left( x \right)} \right)}}{c\left( n \right)}}}$$

where h(x) is the path length for a single isolation tree, E(h(x)) is the average h(x) for datapoint x across the ensemble of isolation trees, and c(n) is the average path length for a dataset of n points, which is used for normalization purposes.

### Modelling and evaluation

Baseline models were trained using only traditional biomarkers to assess the performance improvement when adding novel biomarkers of OS, inflammation and MD. Given that iForest is a black-box model, Depth-based Isolation Forest Feature Importance (DIFFI) was employed to add explainability to the model and identify the most influential predictors.

Experiments were carried out to assess the effectiveness of iForest for the predictive classification task using only traditional features initially, and to assess the change in model performance when adding the additional predictor variables discussed earlier. This created two distinct models for comparison: iForest with traditional features only, and iForest with all predictor features. For comparison, an additional three RF models were also trained using three oversampling techniques, including SMOTE, Borderline SMOTE and ADASYN. To mitigate overfitting due to the small number of positive samples, recursive feature elimination was performed to keep only the top 10 features of the aforementioned 17 biomarkers.

The data was split into training and testing sets using a 70:30 ratio. To assess the stability of the results, 10 iterations of this split were employed and evaluated, and the mean and standard deviations (SD) of the model evaluation metrics were computed, followed by computing the coefficients of variation (SD/mean).

Min–max normalization was applied to all features to scale values within the range [0,1] using the following formula, where X represents each feature:

2 $$X_{scaled} = \frac{{X - X_{min} }}{{X_{max} - X_{min} }}$$

All experiments were carried out in Python 3.11.5. Scikit-learn 1.2.2. was used to implement RF and iForest using default parameters except for the contamination parameter, which is the expected proportion of anomalies in the dataset, and was set to 0.05 representing an expected 5% of G-T2DM in the dataset. All models were assessed using recall, precision, F-1 score and accuracy, which are defined through the following equations:

3 $${\text{Accuracy }} = \frac{{\left( {{\text{TP }} + {\text{ TN}}} \right){ }}}{{\left( {{\text{TP }} + {\text{ TN }} + {\text{ FP }} + {\text{ FN}}} \right)}}$$

4 $${\text{Precision }} = \frac{{{\text{TP}}}}{{{ }\left( {{\text{TP }} + {\text{ FP}}} \right)}}$$

5 $${\text{Recall }} = \frac{{{\text{TP}}}}{{{ }\left( {{\text{TP }} + {\text{ FN}}} \right)}}$$

6 $${\text{F1}}\;{\text{Score }} = { 2} \times \frac{{\left( {{\text{precision}} \times {\text{ recall}}} \right){ }}}{{\left( {{\text{precision }} + {\text{ recall}}} \right))}}$$

where TP represents true positives, FP false positives, TN true negatives and FN false negatives. The mean and SD were computed for the metrics over the 10 iterations for all models.

### Model explainability

To provide much needed explainability to the black-box iForest model, Depth-based Isolation Forest Feature Importance (DIFFI) was added to the analysis pipeline. Described in^54^, DIFFI assigns higher feature importance scores to features that induce higher imbalance in favor of anomalous points, and those that isolate anomalies at shallower depths. Global Feature Importance (GFI) scores are computed by updating cumulative feature importance scores according to the depth of the splitting feature and the induced imbalance at each node, termed the Induced Imbalance Coefficient^54^.

To compute GFI across the 10 iForest models (corresponding to the 10 dataset splits), the scores are aggregated for all models. Features (p) are then ranked in decreasing order according to their cumulative DIFFI score. An additional quantity is then added according to feature rank $\hat{r}$ to further differentiate the most important from the least important features:

7 $$\Delta {\text{GFI}} = 1 - { }\frac{{{\text{log}}\left( {\hat{r}} \right)}}{{{\text{log}}\left( p \right)}}$$

The details of the implementation of global DIFFI computation and feature ranking are provided in the original work by Carletti et al.^54^.

## Results

### Demographic and clinical characteristics

Our data consists of 324 participants divided into two groups according to glycemic outcome, with Tables 1 and 2 presenting categorical and numerical baseline characteristics, respectively. The first group remained as controls (G-Controls), while the second group progressed to T2DM (G-T2DM). Regarding participants in G-Controls, 23.5% (72/307) were in the prediabetic stage (5.5 < BGL < 7 mmol/L) at baseline, while 47.1% (8/17) were prediabetic in G-T2DM. No significant differences were found between G-Controls and G-T2DM in terms of age, gender, hypertension status, smoking, alcohol consumption, cardiovascular disease, and statin use. However, BMI was significantly higher in G-T2DM (p < 0.001).

**Table 1:** Descriptive statistics of the study participants at baseline. Values are described as numbers (%). Significant (p < 0.05) differences were detected using χ^2^ tests.

Characteristic	G-Controls, n = 307	G-T2DM, n = 17	p-value
*Categorical*	*Number (%)*	*Number (%)*	χ^2^
Gender (Female)	178/307 (58.0%)	10/17 (58.8%)	> 0.9
Alcohol	49/307 (16.0%)	3/17 (17.6%)	> 0.9
Smoking	8 /307 (2.6%)	1/17 (5.9%)	> 0.9
Statin Use (Yes)	53/307 (17.3%)	5/17 (29.4%)	0.34
Cardiovascular Disease (Yes)	124/307 (40.4%)	7/17 (41.2%)	> 0.9
Hypertension (Yes)	171/307 (55.7%)	12/17 (70.6%)	0.34
Hypertension Medication (Yes)	106/307 (34.5%)	9/17 (52.9%)	0.2

**Table 2:** Descriptive statistics of the study participants at baseline. Values are described as median (Q1-Q3). Significant (p < 0.05) differences were detected using Wilcoxon rank sum tests.

Characteristic	G-Controls, n = 307	G-T2DM, n = 17	p-value
Numerical	Median (Q1–Q3)	Median (Q1–Q3)	Wilcoxon Rank Sum
Age (years)	66.0 (56.5–74.0)	65.0 (58.0–70.0	0.5
Body Mass Index (BMI) (kg/m2)	26.3 (24.05–29.6)	31.0 (29.0–32.8)	< 0.001
HbA1c (%)	5.8 (5.5–6.0)	6.0 (5.7–6.6)	0.02
Fasting Blood Glucose Level (BGL) (mmol/L)	5.1 (4.6–5.45)	5.4 (5.1–6.1)	< 0.001
Interleukin-10 (IL-10) (pg/mL)	26.17 (16.5–45.9)	15.61 (13.9–21.98)	0.02
Interleukin-6 (IL-6) (pg/mL)	14.91 (9.0–21.7)	24.24 (11.1–42.2)	0.04
Interleukin-1β (IL-1β) (pg/mL)	4.29 (2.505–8.785)	4.72 (2.72–7.79)	0.8
Monocyte Chemoattractant Protein-1 (MCP-1) (pg/mL)	217.31 (180.5–252.9)	226.35 (146.2–259.9)	0.9
8-isoprostane (ng/mL)	1.13 (0.8–2.07)	1.96 (0.8–2.93)	0.4
8-hydroxydeoxyguanosine (8-OHdG) (ng/mL)	138.8 (93.5–193)	143.34 (103.0–188.6)	0.9
C-Reactive Protein (CRP) (mg/mL)	2.0 (1.2–3.1)	1.9 (1.6–3.0)	0.9
Insulin-like Growth Factor (IGF-1) (pg/mL)	275.96 (160.7–376.5)	203.18 (112.5–419.0)	0.9
Oxidized Glutathione (GSSG) (μM)	279.72 (237.5–344.1)	274.87 (216.1–369.8)	0.9
Humanin (HN) (pg/mL)	210.23 (160.0–240.8)	178.46 (146.3–250.33)	0.5
P66^Shc^ (pg/mL)	47.02 (39.7–53.5)	47.572 (37.5–51.2)	0.7
MOTS-c (ng/mL)	546.95 (435.9–680.5)	458.84 (411.3–624.6)	0.4
Complement Component 5a (C5a) (ng/mL)	6.7 (5.4–13.9)	6.56 (4.6–9.43)	0.3
D-dimer (μg/L)	0.37 (0.26–0.55)	0.5 (0.34–0.63)	0.07
Low-Density Lipoprotein (LDL) (mmol/L)	3.3 (2.3–3.8)	3.2 (2.7–3.75)	0.6
High-Density Lipoprotein (HDL) (mmol/L)	1.4 (1.2–1.6)	1.4 (1.2–1.6)	0.7
Total Cholesterol (mmol/L)	5.2 (4.6–5.8)	5.5 (5.2–5.9)	0.2
Triglycerides (mmol/L)	1.2 (0.9–1.7)	1.4 (1.2–2.0)	0.02

### Blood and urinary biomarkers

Table 2 also summarizes inferential statistics on the blood and urinary biomarkers of participants. As expected, the group of participants in G-T2DM had significantly higher baseline levels of HbA1c and BGL. In terms of lipid profile, the two groups were matched except for triglycerides, which was significantly higher in G-T2DM (p = 0.01). Inflammatory biomarkers indicated elevated levels of inflammation in G-T2DM as revealed by significantly higher levels of IL-6 and IL-10. However, the remaining biomarkers of inflammation (MCP-1, CRP, IL-1β, IGF-1) did not reveal any significant differences. No significant differences were found for the mitochondrial biomarkers (HN, MOTS-c, and P66Shc), OS biomarkers (8-isoprostane, 8-OHdG, GSSG) and biomarkers of hemostasis (C5a and D-dimer). However, inflammatory, OS and MD features played a significant role in predicting the risk of T2DM as discussed below.

### iForest performance evaluation and comparison with oversampling techniques

**Figure 3:**
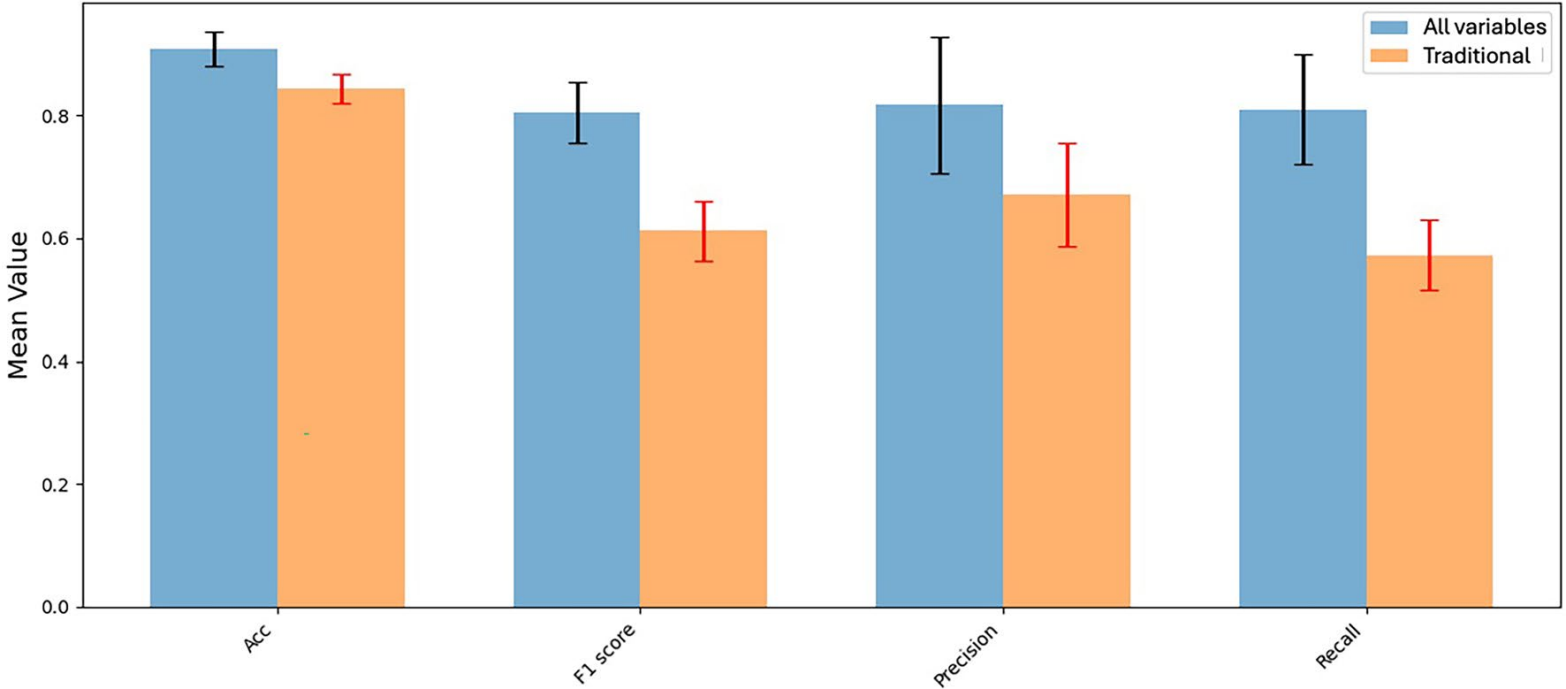
Mean values and standard deviation bars of performance metrics, Accuracy (Acc), F1-score, precision and recall for the two iForest models evaluated across ten folds, one with conventional biomarkers only, and the second using conventional and novel biomarkers of inflammation, OS and MD.

Figure 3 summarizes the mean ± SD of the evaluation metrics obtained across the tenfold cross validation. Augmenting traditional biomarkers of BGL, BMI and LDL with biomarkers of inflammation, OS, and MD improved model performance across all metrics. The biggest performance boost was seen in the model recall, which increased from 0.57 ± 0.06 to 0.81 ± 0.08. Accuracy increased from 0.84 ± 0.02 to 0.91 ± 0.03, F1-score from 0.61 ± 0.05 to 0.81 ± 0.05, and precision from 0.67 ± 0.09 to 0.82 ± 0.11. Accordingly, the coefficients of variation were 3.3% for model accuracy, 9.9% for recall, 13.4% for precision and 6.2% for F1-score. In comparison, the RF models with the oversampling techniques all performed poorly, particularly in terms of precision, which is displayed in Table 3.

**Table 3:** Mean ± SD of evaluation metrics for RF models with three oversampling techniques (SMOTE, SMOTETomek, ADASYN).

Model	Accuracy	Recall	Precision	F-1 score
RF + SMOTE	0.90 ± 0.04	0.40 ± 0.19	0.27 ± 0.11	0.28 ± 0.09
RF + SMOTETomek	0.89 ± 0.08	0.30 ± 0.13	0.22 ± 0.10	0.25 ± 0.11
RF + ADASYN	0.88 ± 0.03	0.46 ± 0.22	0.22 ± 0.06	0.27 ± 0.07

### Feature importance with depth-based isolation forest feature importance (DIFFI)

**Figure 4:**
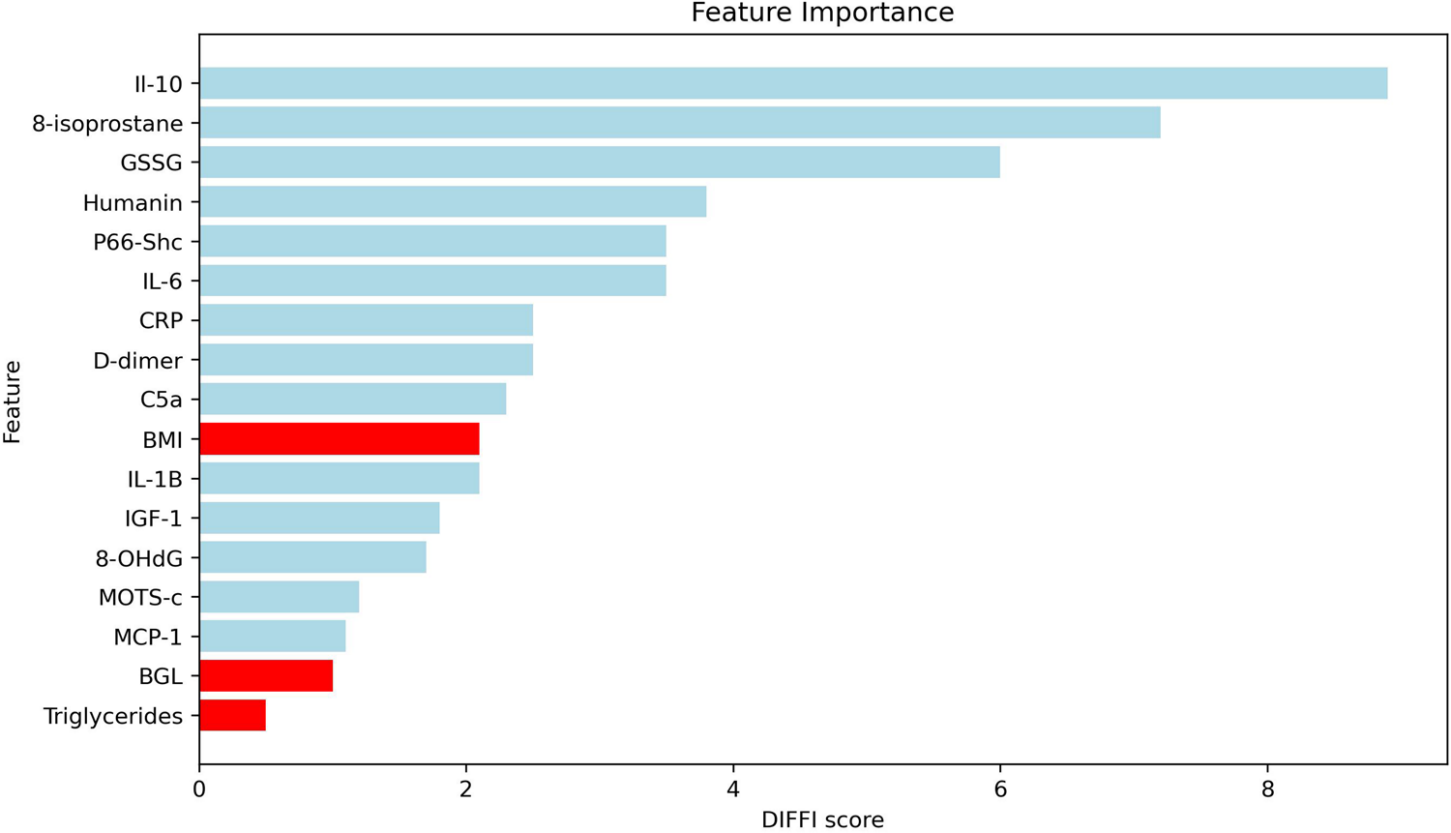
Feature importance as measured by DIFFI scores. The red features are traditional features (BGL, BMI and triglycerides), while the blue features are the novel features considered in this study (OS, inflammation and MD).

Global feature importance scores obtained through DIFFI are summarized in Fig. 4. The five most important features were IL-10, 8-isoprostane, GSSG, HN and P66Shc. Traditional biomarkers of BGL and triglycerides were the least important features overall, whereas BMI was only in 10th place out of 17 features.

## Discussion

The objective of this study was to explore the performance of biomarkers of OS, inflammation, and MD for the prediction of T2DM occurrence with a highly imbalanced dataset utilizing iForest, an anomaly detection algorithm. By leveraging DIFFI scores, explainability was achieved for the black box model, and important features for the prediction were identified that can be of clinical value for selecting appropriate treatment.

According to glycemic outcome, participants were divided into two groups: Those remaining as controls (G-Controls) and those progressing to T2DM (G-T2DM). One-quarter of participants in the control group were prediabetic at baseline, and more than one-third of those developing T2DM were not prediabetic. This highlights the need for measures beyond BGL to better monitor and predict the development of this disease.

The dataset used in this study suffered from a class imbalance (ratio < 1:15), with those presenting with T2DM as the minority class at only 5.5% of participants. This imbalance was addressed by utilizing anomaly detection rather than traditional binary ML techniques. iForest models were trained with two sets of features for predicting the risk of T2DM development. One set consisted of only traditional biomarkers (BGL, BMI, triglycerides), and the second included both traditional and new biomarkers (OS, inflammation and MD). Additionally, the iForest method was compared with various oversampling techniques to assess the utility of OCC in the presence of a small sample of positive cases for model training.

Baseline BMI, IL-6, and IL-10 were significantly higher for participants in G-T2DM. IL-6 is an inflammatory cytokine that was previously found to be increased in individuals with T2DM^55^, and increased levels of IL-6 in adipose tissue have been linked to insulin resistance^56^. In obese individuals, the release of non-esterified fatty acids from adipose tissue is believed to contribute to insulin resistance and β-cell dysfunction, with the term diabesity coined to illustrate the tight association between obesity and T2DM^57,58^. However IL-6 can also have an anti-inflammatory effect and improves glucose metabolism^59,60^. Hence, models that are based on single features may not identify the complex feature interaction. Activity of IL-6 may be further concentration dependent, which activates different signaling pathways including reactive oxygen specifies reduction^61^.

iForest models outperformed RF with oversampling across all metrics except for accuracy, however, this was due to the latter models’ bias towards predicting the negative class. These results indicate the advantage of employing the OCC technique in the case of data scarcity, particularly when the features of interest are not routinely collected or are expensive to obtain^33^.

The inclusion of biomarkers of OS, inflammation and MD improved the performance across all metrics in comparison to predictive modelling with only traditional biomarkers of BGL, BMI and triglycerides. The greatest boost in performance was observed for recall and F1-scores. This is particularly important given the higher cost of missing future cases of T2DM as opposed to predicting false positives, considering that interventions mainly consist of lifestyle changes. Furthermore, the coefficients of variation for the evaluation metrics indicated low to moderate variability, with values below 10% for accuracy, F-1 score, and recall indicating good stability for the trained model.

The top five predictors in terms of DIFFI scores were IL-10, 8-isoprostane, GSSG, HN and P66Shc, while the lowest scores were obtained by BGL and triglycerides, further highlighting the potential role of these novel biomarkers for ML prediction of T2DM development.

The anti-inflammatory IL-10 is generally hypothesized to play a protective role in T2DM^62^, and IL-10 gene polymorphisms have been suggested for T2DM screening^63,64^. IL-10 is believed to improve glycemic control through its immunomodulatory effects by inhibiting cytokine production^65^. This is in line with the results of our study, where significantly lower levels of IL-10 were observed in G-T2DM.

8-isoprostane, a biomarker of lipid peroxidation, has shown varying efficiency as a biomarker for prediabetes^10,66^ . However, the present study agrees with the results reported by Schöttker et al.^67^, in which higher levels of 8-isoprostane were associated with the incidence of T2DM in participants 65 years of age or older. Given that the median age for patients developing T2DM in our study is 65, the reduced tolerance for OS with age would also be apparent.

GSSG is the oxidized form of GSH, an antioxidant defense system primarily stored and released from erythrocytes^68^. GSH is converted to GSSG in the presence of free radicals, and in individuals with T2DM, regeneration of GSH from GSSG is impaired because of insufficient factors necessary for this conversion. Furthermore, increases in free radical production as part of T2DM progression associated with increased BGL, in turn, activates the GSH scavenger, producing higher levels of GSSG^56^. Hence, the combined action of meta-inflammation and GSH antioxidant activity indicates the interactive role of diverse biomarkers in possibly mitigating disease progression that can lead to a novel treatment pathway for T2DM in conjunction with traditional clinical options.

HN is a MDP that plays a key role in metabolism and insulin sensitivity^69,70^. Voight and Jelinek^8^ found decreased levels of HN in prediabetic patients, given that HN has an important role in glucose metabolism through its antiapoptotic and antioxidant functions^71^. Conversely, P66Shc, a Shc protein that modulates OS and promotes apoptosis, has been implicated in T2DM development and progression through its association with pancreatic β-cell dysfunction and suppression of insulin signaling^72,73^.

Our results indicate important interactions between inflammatory and OS biomarkers associated with T2DM progression over time and highlight the lesser role of traditional features. To gain a better understating of the specific interactions between these biomarkers a larger number of participants is required in order to obtain performance metrics and feature importance scores that increase the reliability of our results. Furthermore, a larger dataset would allow for appropriate hyperparameter tuning to be carried out to optimize the results further. Additionally, the possible change in data distribution introduced by missing data imputation may have impacted subsequent ML pipelines and feature importance. Finally, due to data scarcity, the selected cohort included all participants without T2DM, which should be investigated in future studies with the availability of a larger and more specific cohort to provide targeted insights.

## Conclusion

Based on the results of this study, various conclusions can be inferred. First, typical monitoring of T2DM risk through BGL may not provide a comprehensive picture of T2DM disease progression. Influential biomarkers identified were IL-10, 8-isoprostane, GSSG, HN and P66Shc, revealing the potential for biomarkers of inflammation, OD and MD to serve as a guide for targeted, personalized intervention in the prevention of T2DM incidence.

### Supplementary Information

Supplementary Information.

## Data Availability

Data is made available to readers with relevant interests by contacting Dr. Herbert Jelinek (herbert.jelinek@ku.ac.ae).

## Abbreviations

8-OHdG: 8-Hydroxydeoxyguanosine
ANN: Artificial neural network
BGL: Blood glucose levels
C5a: Complement component 5a
DIFFI: Depth-based Isolation Forest feature importance
ECD: Early Classification Diabetes
HN: Humanin
iForest: Isolation forest
IGF-1: Insulin-like growth factor-1
IL-1β: Interleukin-1β
IL-6: Interleukin-6
IL-10: Interleukin-10
GSH: Reduced glutathione
GSSG: Oxidized glutathione
MCP-1: Monocyte chemoattractant protein-1
MD: Mitochondrial dysfunction
MDP: Mitochondrial-derived peptides
ML: Machine learning
MOTS-c: Mitochondrial open-reading-frame of the twelve S rRNA-c
OCC: One class classification
OS: Oxidative stress
PIDD: Pima Indians Diabetes Dataset
SHAP: Shapley additive explanations
SMOTE: Synthetic minority oversampling technique
SVM: Support vector machine
T2DM: Type II diabetes mellitus
