# Supplementary material for: Exploratory risk prediction of type II diabetes with isolation forests and novel biomarkers
Source: Sci Rep. 2024 Jun 22;14:14409. doi: 10.1038/s41598-024-65044-x (PMC11193708; doi:10.1038/s41598-024-65044-x)
Supplement: Supplementary file 1 — Supplementary Information. [file 41598_2024_65044_MOESM1_ESM.docx]

Supplementary Material


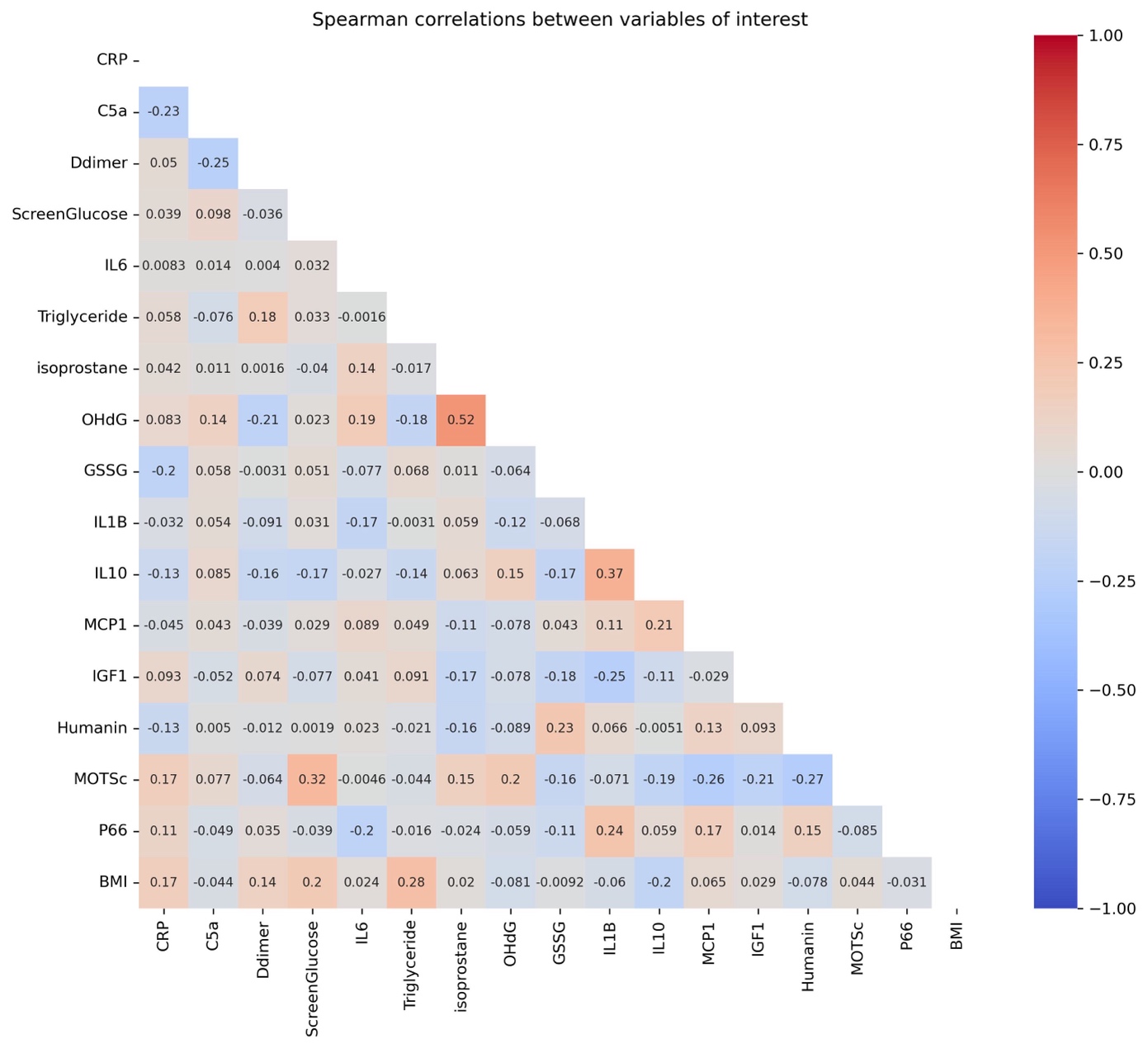


**Figure S1.** Spearman correlations between the variables of interest reveal only weak correlations.
